# Investigation of the positivity profile for the skin prick test in children infected with parasites in the metropolitan region of Pernambuco, Northeast of Brazil

**DOI:** 10.1186/1939-4551-8-S1-A241

**Published:** 2015-04-08

**Authors:** Victor Torres Teodósio, Wheverton Correia Do Nascimento, Cássia Oliveira Nóbrega, Tiago Elias Melo, Georgia Araujo, Érica De Souza Fernandes, Juliana Prado Gonçales, Décio Medeiros, Constança Simões Barbosa, Valdênia Oliveira De Souza

**Affiliations:** 1 Research Center Aggeu Magalhães - Fundação Oswaldo Cruz, Brazil; 2 Federal University of Pernambuco, Brazil

## Background

Parasitic infections induce Th2 cell immune profile and modulate the symptoms of asthma and rhinitis. The objective is to analyze the modulation of parasitic infection in the skin prick test profile in children with asthma from two localities in the metropolitan region of Recife - Pernambuco.

## Methods

Children between 2 and 14 year old were submitted to a parasitological survey using the Hoffman, Pons e Janer (3 blades) method and the Kato-Katz (2 blades) method. The ISAAC questionnaire was applied to collect informations related to asthma. The Skin Prick Test was applied using extracts of *Dermatophagoides pteronyssinus* (DP), *Blomia tropicalis* (BT), *Blatella germânica* (BG), *Periplaneta americana* (PA), fungus and cat epithelium (EPC).

## Results

Sixty-two children were registered, 35 (65,17%) classified as having asthma by ISAAC. Between asthmatics 11 (31,42%) were infected with parasites and 24 non-infected (68,58%). Among non-asthmatic children (n=27, 43,54%), 25 were infected (92,6%) and 2 non-infected (7,4%). The parasites identified in asthmatic children were *Enterobius vermicularis* (n=2, 18,18%), *Ascaris lumbricoides*, *Trichuris trichiura and Ancylostoma sp* (n=1, 9,09%), *Giardia lamblia* (n=5, 45,45%), *T. trichiura* and *Ancylostoma sp* (n=1, 9,09%), *T. trichiura and A. lumbricoides* (n=1, 9,09%), *T. trichiura* (n=1, 9,09%); within the non-asthmatic were *A. lumbricoides* (n=3, 12%), *G. lamblia* (n=10, 40%), *T. trichiura* (n=6, 24%), *Schistosoma mansoni* (n=1, 4%), *Ancylostoma sp* (n=2, 8%), *S. m*ansoni, *T. trichiura* e *Ancylostoma sp* (n=1, 4%), *T. trichuris*, *A. lumbricoides* and *Ancylostoma sp* (n=2, 8%). The prick test was positive in 18 children (29,03%), with 7 infected (11,29%) and 11 not infected (17,74%). The positivity profile of the prick test were 2 children only for DP (11,11%), 1 for BT (5,55%), 11 for DP and BT (61,11%), 1 for DP, BT and fungus (5,55%), 1 for DP, BT, BG, PA and EPC (5,55%), and 1 for BT,BG and PA (5,55%).

## Conclusion

The parasitic infection was more frequent in non-asthmatic children. It was not possible to verify alterations in the positivity of the prick test among asthmatic children and the association with parasitic infections. The most frequent allergens in the positivity of the prick test was *Dermatophagoides pteronyssinus* and *Blomia tropicalis.*

